# Correlative Raman and immunofluorescence imaging reveals different protein abundance between stress granules induced by oxidative damage☆

**DOI:** 10.1016/j.jinorgbio.2025.113091

**Published:** 2025-09-29

**Authors:** Kevin L. Gery, Sashary Ramos, Jennifer C. Lee

**Affiliations:** Laboratory of Protein Conformation and Dynamics, Biochemistry and Biophysics Center, National Heart, Lung, and Blood Institute, National Institutes of Health, Bethesda, MD, USA

**Keywords:** Sodium arsenite, Hydrogen peroxide, ROS, G3BP, TDP-43, eIF4G

## Abstract

Heavy metal toxicity generates reactive oxygen species (ROS) that can contribute to neurodegeneration. Oxidative damage from exposure to metals such as sodium arsenite will activate the integrated stress response and may result in the cytosolic formation of stress granules (SGs), which have been implicated in neurodegenerative disorders such as amyotrophic lateral sclerosis. Here, two different ROS sources, sodium arsenite and hydrogen peroxide, under acute (1 h) and chronic (24 h) conditions, were used to induce SG formation in human osteosarcoma (U-2 OS) cells and investigate if characteristics of SGs could depend on the induction. Specifically, correlative Raman and immunofluorescence imaging (CRIFI) was developed to evaluate the relative protein abundance found in SGs to ascertain their potential as loci for protein accumulation. Interestingly, while there are differences in the punctate-staining phenotypes for different stressors, two types of puncta visualized by CRIFI were common to all treatment conditions, where notably a subset exhibited protein concentration above cytosolic background, indicating that only some SGs are composed of protein-rich, dense phases. Differences in protein abundance between SGs were also observed within a single cell, suggesting that individual SGs can develop differently. These results demonstrate the versatility and the strength of pairing Raman spectroscopy, which allows for probe-free detection of different chemical functional groups, with specific protein localization granted by immunofluorescence, providing new cellular insights unattainable by either modality alone.

## 1. Introduction

Heavy metal toxicity is an established source of cellular stress that can contribute to the development of neurodegenerative disorders. One such metal is arsenic, an environmental contaminant, which is produced by several industrial processes [1]. Arsenic toxicity has been shown to cause neurodegeneration [1-8]. On a cellular level, exposure to arsenite, the trivalent (+3) state of arsenic, results in the generation of reactive oxygen species (ROS) either by direct reduction [9-11] or by interfering with the cellular antioxidant defense system [10,12]. In neurons, oxidative damage biochemically contributes to degeneration [13,14], and some neurodegenerative disease models are generated by compounds that invoke oxidative stress [15-17]. ROS buildup is also observed in age-related diseases [13].

Under stress conditions, cells may respond by forming transient, phase-separated structures known as stress granules (SGs). The proposed method by which SGs alleviate stress is the selective sequestration of halted translation machinery and mRNA transcripts, providing a confined environment for greater discretion of specific expression levels and preventing non-essential protein synthesis [18-22]. SGs can also indirectly aid in reduction of ROS by confining cofactors that ordinarily inhibit antioxidant enzymes [23]. In mammalian cells, acute sodium arsenite (NaAsO_2_) treatment has long been established as the standard method for robust induction of SGs, reiterating the link between heavy metals and the mechanisms of neurodegeneration. Chronic stress by sodium arsenite treatment has also been reported to induce SGs [24,25]. Although sodium arsenite is the most prevalent stressor for SG induction in cell culture, there are other compounds that introduce ROS that have been reported to induce SGs, including direct introduction by hydrogen peroxide (H_2_O_2_) treatment [26]. Beyond oxidative stressors, SGs can also be induced by osmotic [27], proteostatic [28,29], viral [30], nutrient [31], hypoxic [32], and heat stress [33,34]. Moreover, SGs can form in the complete absence of stress by the overexpression of select SG proteins alone [35,36]. The protein composition of SGs and which proteins are required for their formation can vary based on the precise dangers posed to the cell [19] as is the case with induction by H_2_O_2_, in which SGs can form without the canonical phosphorylation of eukaryotic initiation factor 2^α^ (eIF2^α^) and, once formed, contain lower levels of several other proteins, including eukaryotic initiation factor 4G (eIF4G) [26,37].

Aside from identification of individual protein species, the macromolecular distribution within SGs is also of interest [38-40]. Previous studies have suggested that SGs may be low-density condensates with insignificant protein enrichment [39,40], which is an interesting observation given that, although often driven by RNA binding [39-42], proteins are the key factor in phase separation. Therefore, in order to achieve an isolated phase, proteins should inherently be in greater local abundance within SGs than in the surrounding cytosol. Determining whether or not this is the case is relevant in the context of a relation between SG-proteins and disease; importantly, SGs are also found alongside protein aggregates in affected tissues of patients afflicted with neurodegenerative disorders [43], and a significant portion of SG-associated proteins, especially RNA-binding proteins (*e.g.*, transactive response DNA binding protein of 43 kDa (TDP-43), heterogeneous nuclear ribonucleoprotein A1 (hnRNP A1), fused-in sarcoma (FUS), and T-cell intracellular antigen 1 (TIA-1)), are directly implicated in disorders such as amyotrophic lateral sclerosis [43-46]. As loci for the collection of aggregation-prone proteins, SGs have been theorized to play a role in contributing to pathological amyloid formation; however, how this might occur and under what specific conditions remain unknown. Thus, it is essential to evaluate the protein abundance inside SGs and to monitor how they vary with different stressors and timeframes to determine a possible disease connection.

In this work, we present a correlative microscopic imaging method to characterize SGs in cells that allows us to localize endogenous target proteins using immunofluorescence (IF) and map the molecular composition of their environment by Raman spectral imaging (RSI). RSI is a probe-free technique that can yield spatially resolved data on molecular vibrations. It is well suited for cellular imaging as it is easily adaptable to any standard microscope, uses visible laser light, and its rich spectral features reflect the type of biomacromolecules (*e.g.* proteins, lipids, and nucleotides) present at any given point and inform on protein secondary structure [47,48]. RSI therefore enables us to assess whether SGs are truly protein-rich or not. To afford both classification and visualization of target species within the complex cellular milieu, we combined RSI with IF, which we call correlative Raman and immunofluorescence imaging (CRIFI).

Using CRIFI, we visualized SGs induced by oxidative stress caused by two chemical stressors, sodium arsenite and hydrogen peroxide, in human osteosarcoma (U-2 OS) cells under acute (1 h) and chronic (24 h) conditions. To our knowledge, there is one previous SG study that coupled fluorescence with RSI by Shibuya et al. [38], where a near-infrared fluorescent protein (iRFP) fusion of Ras GTPase-activating protein-(SH3 domain)-binding protein (G3BP) was transiently expressed in HEK293A cells. G3BP is a protein that is commonly used to identify SGs [35,39,42] because both of its isoforms, G3BP1 and G3BP2, contribute to SG formation [36] and are considered necessary for SG formation *in vivo* [36,39,42]. While the use of an FP-tagged protein is efficient and facilitates live-cell imaging, we chose the broader approach of immunofluorescence because we sought to study endogenous SGs without overexpression effects so as not to perturb observation of induction-based differences. Indeed, it has been shown that purified G3BP1 alone can nucleate SG formation from cell lysates *in vitro* [41]. Additionally, CRIFI allows the flexibility to localize additional proteins-of-interest (*e.g.* eIF4G [19,26,49] and TDP-43 [43,50]), which we demonstrate with two fluorophores (Alexa405 and Alexa647) that have distinct excitation windows with energies that do not include the Raman excitation wavelength (488 nm). Based on the number of puncta-positive cells and the number of puncta per cell, characteristic phenotypes are observed for each of the four treatments. Interestingly, a common phenotype is seen by CRIFI for all treatments in which only a subpopulation of the G3BP1-positive puncta generated by any one treatment exhibits protein accumulation above cytosolic background. This was an unexpected result given that SGs were known to vary from one inducer to another, but not under the same induction, suggesting they may exhibit characteristics that IF cannot discern. CRIFI also enabled evaluation of protein abundance differences between SGs within a single cell, an observation not previously reported. Collectively, these results elicit a need for complementary methods to classify SGs in cells beyond the currently accepted SG-markers; the ability to evaluate SG compositions could be especially valuable in revealing their potential differences with respect to disease progression.

## 2. Results

### 2.1. Biochemical assays on NaAsO_2_ and H_2_O_2_ treatments

While SG formation under nonlethal stress conditions is well established for acute (1 h) sodium arsenite (500 μM) treatment of U-2 OS cells, chronic (24 h) treatment is not as common. Furthermore, the concentration of H_2_O_2_ needed for SG induction is unclear. Thus, we used IF to determine the specific concentrations of NaAsO_2_ for 24 h and H_2_O_2_ for both 1 and 24 h treatments for our cellular imaging study (Fig. S1). Based on these IF images (Fig. S1), we chose the following four treatment conditions: [NaAsO_2_] = 500 and 35 μM; [H_2_O_2_] = 700 and 300 μM for acute and chronic stress, respectively.

**Fig. 1.**
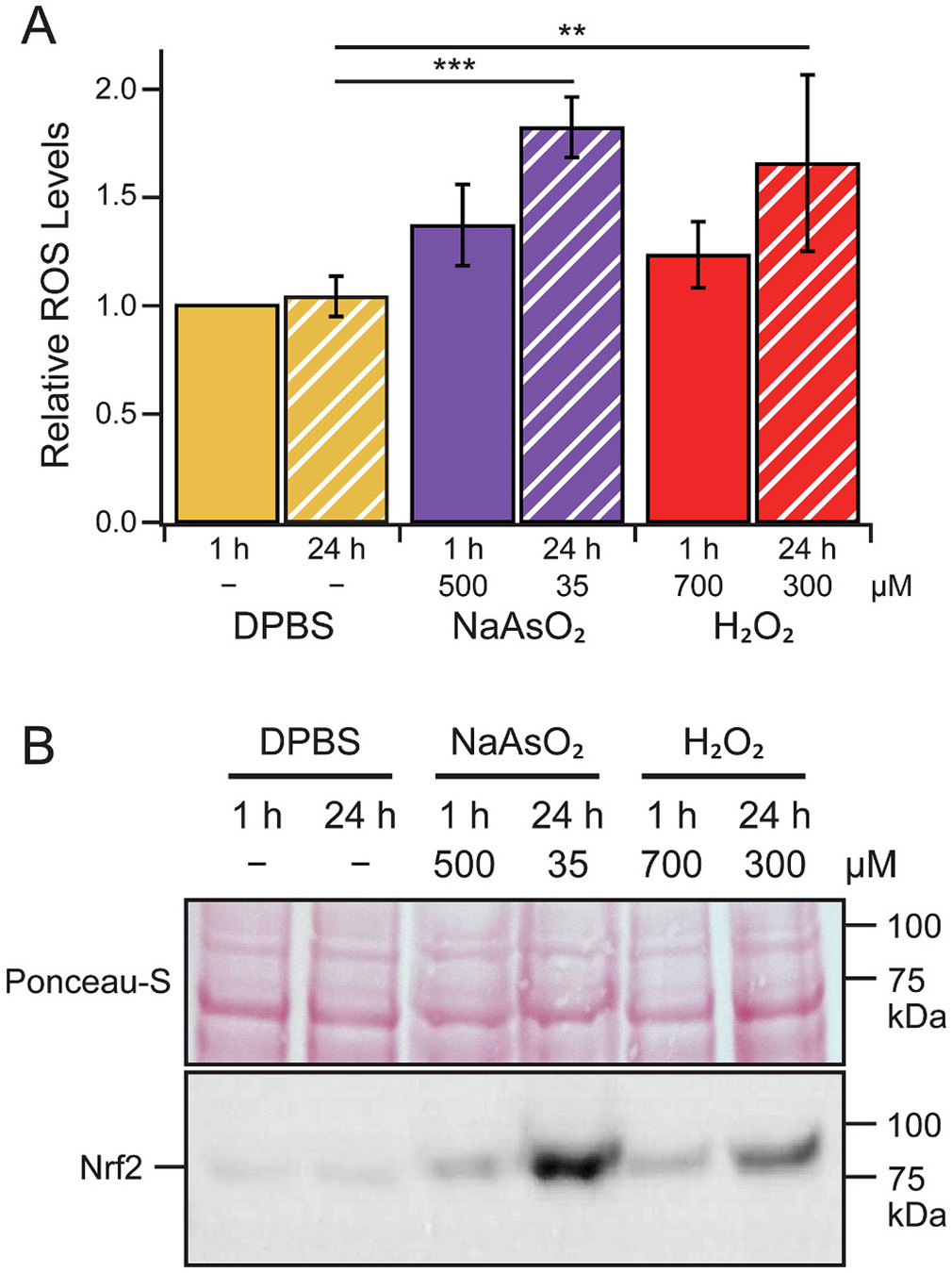
Chemical stressors produce oxidative damage in U-2 OS cells. (A) Quantification of ROS levels in untreated (DPBS) or stressed U-2 OS cells with additions of NaAsO_2_ or H_2_O_2_ determined by the DCFH-DA assay. Concentration and duration are as indicated. Relative ROS level is determined by correcting raw fluorescence counts by protein concentration and dividing by the control DPBS value. Error bars represent 95 % CI (*n* = 5 biological replicates with 3 technical replicates for each sample). *p* values calculated by one-way ANOVA (** and *** represent *p* < 0.01 and *p* < 0.001, respectively). (B) Comparison of Nrf2 antioxidant transcription factor levels by western blot analysis. Ponceau S-stained blot after transfer is also shown above. Full blot images are shown in Fig. S2.

Next, biochemical assays were conducted to quantify ROS accumulation in cells exposed to these selected treatments. Both chronic treatments of NaAsO_2_ and H_2_O_2_ resulted in the increased production of ROS in U-2 OS cells as determined by a standard dichloro-dihydro-fluorescein diacetate (DCFH-DA) assay, monitoring the oxidation of DCFH-DA into fluorescent dichloro-fluorescein (DCF) (Fig. 1A). The observed increases were confirmed to be a result of the applied chemical stressors, as the control cells at 24 h showed no changes in ROS levels.

ROS can initiate different antioxidant pathways as a stress response, not all of which involve the formation of SGs [51]. A common observation in the presence of oxidative stress is the inactivation of Kelch-like ECH-associated protein (Keap1), a suppressor of nuclear erythroid 2-related factor 2 (Nrf2), which in turn initiates transcription of antioxidative stress genes. This pathway has been reported to be active in arsenite stress [52,53]. To verify that oxidative damage had occurred, Nrf2 levels were compared by western blot analysis as a readout of antioxidant pathway induction (Fig. 1B). In each stress condition, observed increases in Nrf2 protein levels indicate that ROS levels were sufficiently damaging to the cell to activate a stress response. In congruence with the ROS measurements, the increase in Nrf2 expression was greater for both chronic treatments than in the corresponding acute conditions, indicating that the cellular response to chronic stress was proportionately increased.

### 2.2. NaAsO_2_ and H_2_O_2_ induce distinct SG-puncta phenotypes

**Fig. 2.**
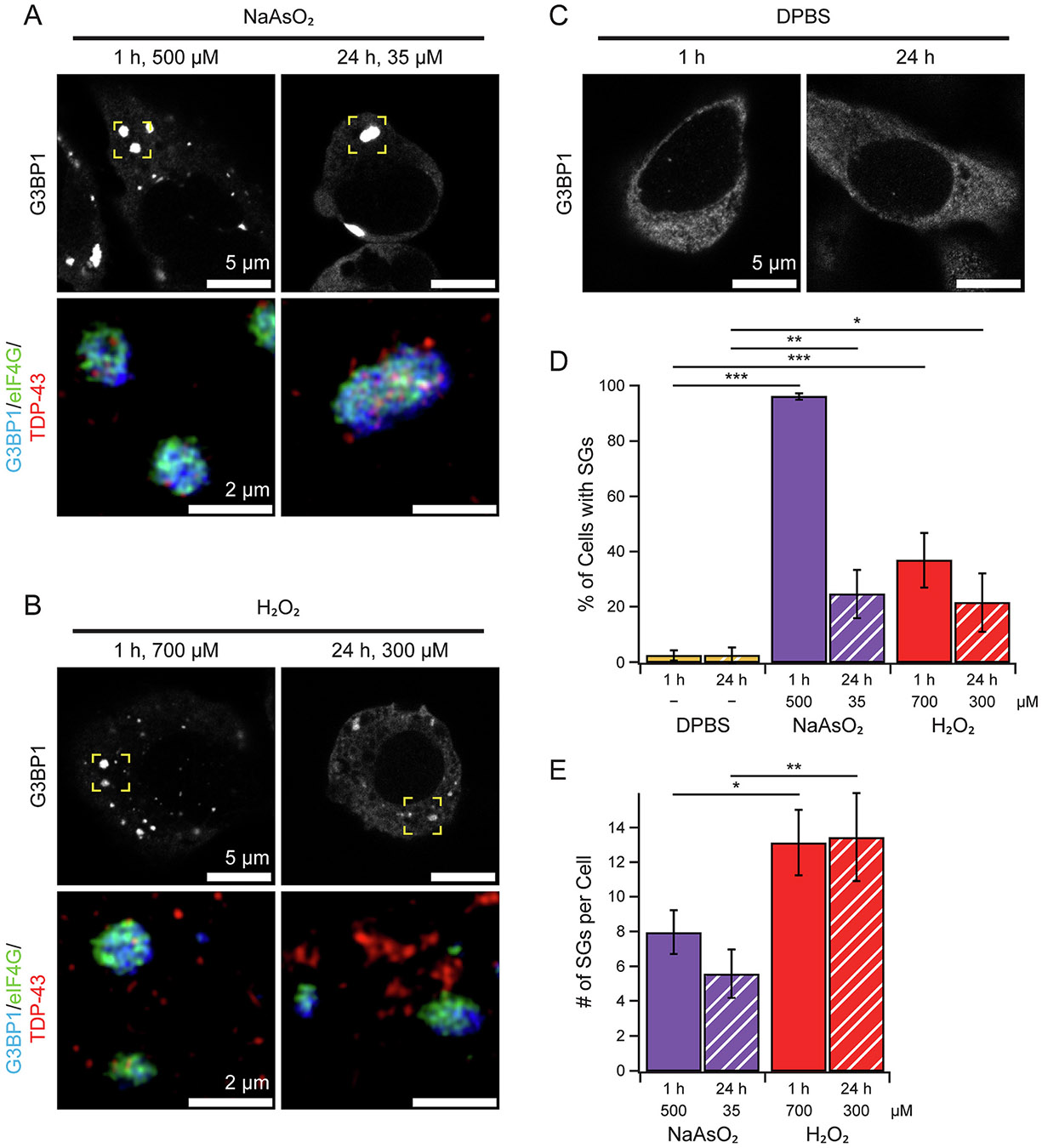
Stress granules are induced under oxidative stress. Immunofluorescence images of SG-associated proteins, G3BP1, eIF4G, and TDP-43 in U-2 OS cells treated with NaAsO_2_ (A) or H_2_O_2_ (B). Concentration and duration are as indicated. (C) Untreated control cells. Anti-G3BP1 (WH0010146M1) is detected with secondary goat anti-mouse labeled with Alexa405, and anti-eIF4G (PA5–83016) and anti-TDP-43 (ab133547) are conjugated with CF488 and CF647 dyes, respectively, shown as a composite with anti-G3BP1 (bottom). Yellow boxes in the top row indicate regions imaged with a super resolution Airyscan 2 detector. Scale bars as indicated. (D) Number of cells per 100 cells (%) for each solution condition that presented with SGs as determined by the presence of G3BP1-positive puncta (*n* = 3 biological replicates, total of 300 cells were counted). (E) Average number of SGs per SG-positive cell using G3BP1 as the marker. Representative sample images used for counting are shown in Fig. S3. Error bars represent 95 % CI (*n* = 3 biological replicates). *P* values calculated by one-way ANOVA (*, **, and *** represent *p* < 0.05, *p* < 0.01, and *p* < 0.001, respectively).

All stress treatments used in this experiment produced puncta staining positive for G3BP1 in U-2 OS cells as well as other SG-associated proteins TDP-43 and eIF4G (Fig. 2A and B bottom panels). Control cells are shown in Fig. 2C. The colocalization of multiple SG proteins that are only present in matured SGs [54] strongly supports that the punctum is a SG and not simply condensation or aggregation of any one component. Interestingly, some of the larger puncta, which display evenly distributed G3BP1, can be resolved at super resolution to exhibit partitioning of TDP-43 into smaller puncta scattered across a single punctum (Fig. 2A, bottom right panel), an observation which has also been recently reported [55]. Distinctive distribution of eIF4G is also seen within the G3BP1 footprint as reported for other core proteins such as poly(A)-binding protein 1 (PABP-1) [56].

As expected, the standard acute treatment of arsenite (500 μM) produced G3BP1-puncta in nearly every cell, markedly more than any of the other conditions (Fig. 2D and S3). Although chronic NaAsO_2_ and both H_2_O_2_ treatments were less effective at inducing puncta in total cells, it is interesting to note the comparably higher degrees of oxidative damage recorded for them (Fig. 1). This inverse correlation does not, however, indicate that SG formation primarily occurs under less intense oxidative damage as both chemical stressors at lower concentrations were insufficient to induce a puncta phenotype (Fig. S1). Further comparison can be drawn between the G3BP1-puncta phenotypes within cells (Fig. 2E). While H_2_O_2_ was overall less efficient at producing SG-positivity in total cell counts, the resulting individual SG-positive cells had more SGs compared to that of either NaAsO_2_ treatments. Puncta in H_2_O_2_-treated cells also appear smaller, suggesting they may differ from the arsenite-induced puncta (Fig. 2A, B, and S3).

### 2.3. Correlative Raman and immunofluorescence imaging (CRIFI)

**Fig. 3.**
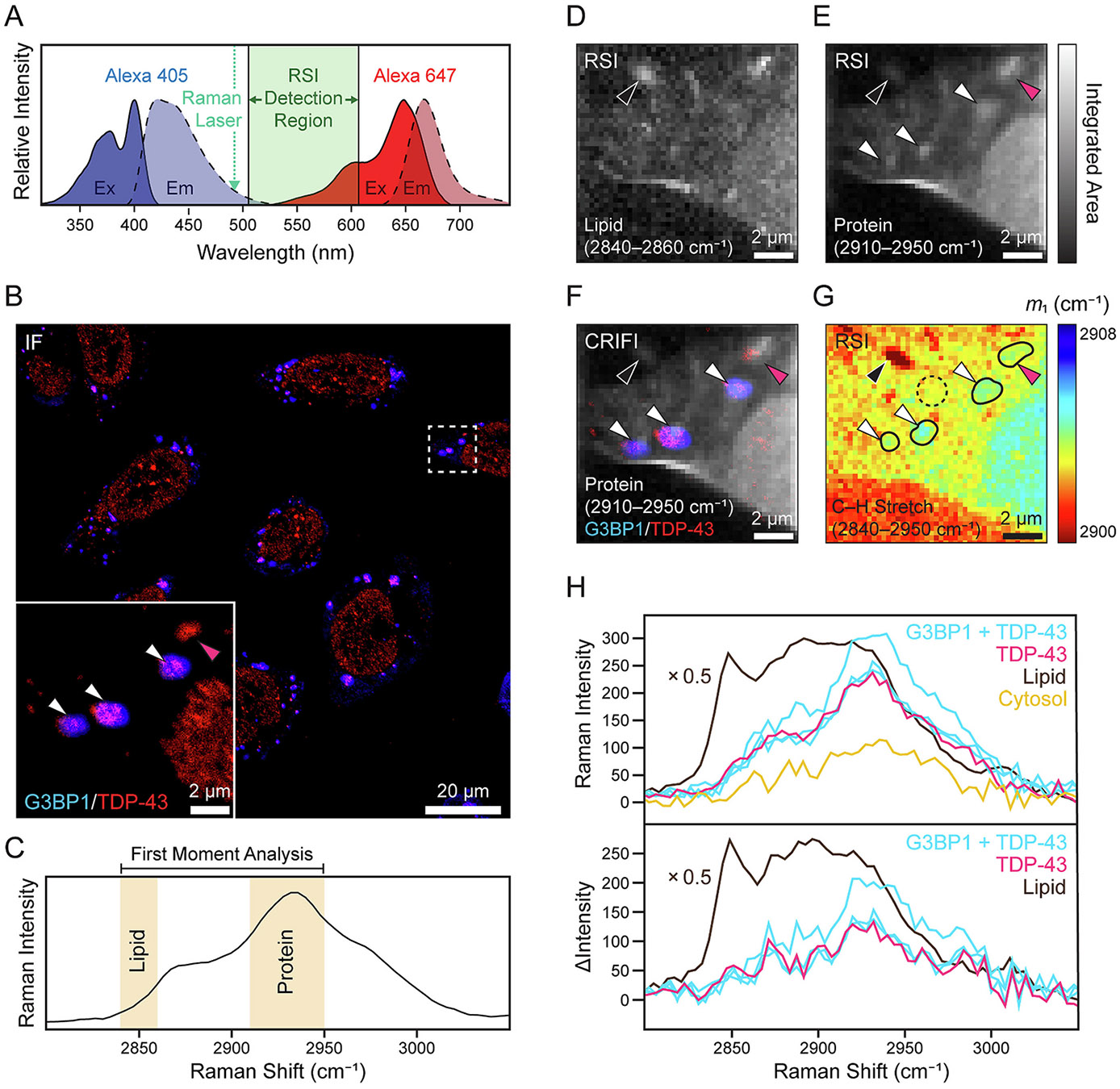
Correlative Raman and immunofluorescence imaging (CRIFI). (A) Fluorescence excitation and emission spectra of goat anti-mouse secondary antibody labeled with Alexa405 (blue) and goat anti-rabbit secondary antibody labeled with Alexa647 (red). Vertical dashed line indicates the wavelength of the Raman laser (488 nm). (B) Immunofluorescence image of U-2 OS cells stressed for 1 h with 500 μM sodium arsenite. Anti-G3BP1 (WH0010146M1) and anti-TDP-43 (MA5–35273) antibodies are detected using the Alexa405- and Alexa647-labeled secondary antibodies, respectively, for which spectra are shown in panel A. Expanded view of the dashed box is shown as an *Inset*. Scale bars as indicated. (C) Representative Raman spectrum of C─H stretch region for an entire cell after background subtraction. Spectral ranges used for integration to generate respective Raman maps are shaded and labeled. Correlated Raman maps of lipid (D) and protein (E) content in the boxed region in panel B were collected using a 1.5 NA 60× oil immersion objective with 100-ms accumulation time per pixel with 250-nm step per pixel using 100-mW laser power. (F) Composite IF and RSI image of the G3BP1/TDP-43 inset in panel B and the protein map in panel D. (G) First moment $\left(m_{1}\right)$ analysis of the C─H stretch across the specified frequency range. RSI maps use the same grayscale for their respective minimum/maximum integrated areas. White and magenta arrowheads indicate colocalization positions of increased protein content with fluorescent puncta positively labeled for either both G3BP1 and TDP-43 or TDP-43 alone, respectively, and black arrowhead indicates a lipid-rich punctum. Scale bars as indicated. (H) Averaged Raman spectra across ROIs shown in panel G. Traces correspond to ROIs which stained for both G3BP1 and TDP-43 (blue, white arrowheads) and for only TDP-43 (magenta, magenta arrowhead), have high lipid signal (brown, black arrowhead), or cytosolic background (yellow, dashed circle). The intensity of the lipid punctum spectrum is scaled by a factor of 0.5. Raman spectra after buffer subtraction (top). Difference spectra (bottom) after subtracting the cytosol (yellow) from each of the traces.

To perform CRIFI, fluorophores are selected such that excitations are at energies higher (405 nm) and lower (647 nm) than that of the Raman excitation laser (488 nm) (Fig. 3A) so as to have no spectral excitation overlap. Even minimal excitation by the Raman laser of fluorophores that emit into the Raman detection window will produce a strong background, prohibiting analysis. Herein, U-2 OS cells are treated under specified solution conditions, fixed, and labeled with two antibodies by standard methods. SGs are first identified under fluorescence microscopy (confocal or widefield) based on antibody staining, then mapped by RSI.

An example CRIFI data set is shown in Fig. 3 for an acute arsenite treatment which produced a strong punctate phenotype for both G3BP1 (blue) and TDP-43 (red) (Fig. 3B). Here, IF images are first collected using an Evident FV4000 confocal laser scanning microscope before transferring to the Raman microscope to perform RSI on the same cell. Raman spectral bands for methylene (CH_2,_ 2840–2860 cm^−1^) and methyl (CH_3_, 2910–2950 cm^−1^) stretches (Fig. 3C) were integrated to generate lipid (Fig. 3D) and protein (Fig. 3E) maps, respectively. In the lipid map, a lipid-rich structure can be discerned (denoted by a black arrowhead), and, in the protein map, there exist multiple puncta that exhibit increased protein content (denoted by white arrowheads) colocalizing with the puncta positive for both SG-markers as shown in the composite CRIFI image (Fig. 3F). It is interesting to note the correlation of a protein-rich punctum (denoted by a magenta arrow) with a TDP-43 positive foci, which is devoid of detectable G3BP1. This suggests that under this stress condition, TDP-43 can accumulate outside of canonical SGs containing G3BP1.

While methylene and methyl stretches are more predominant in lipids and proteins, respectively, they are not exclusive and have some overlap with each other as well as nucleotides. Thus, we sought to use a model-free analysis of the C─H stretching region to distinguish these biomacromolecules and determined that a first-moment analysis ($m_{1}$, which represents weighted mean frequency) of a portion of the C─H stretching region (2840–2950 cm^−1^) would suffice. The resulting $m_{1}$ Raman map, shown in Fig. 3G, reveals the locations of the four protein-rich puncta (outline in black), distinguishable from their cytosolic surroundings. Notably, they are shifted to higher frequencies relative to the cytosol, and lipid-puncta are shifted to lower frequencies, in accord with the relative abundance of methyl to methylene stretches [57] (Fig. 3C). This analysis readily affords the identification and classification of protein- and lipid-puncta in a single Raman map and is used hereinafter. By comparing Raman spectra of the regions identified by said analysis to the cytosolic background, protein accumulation is clearly evident (Fig. 3H).

### 2.4. CRIFI reveals differences in protein levels in G3BP1-positive puncta

**Fig. 4.**
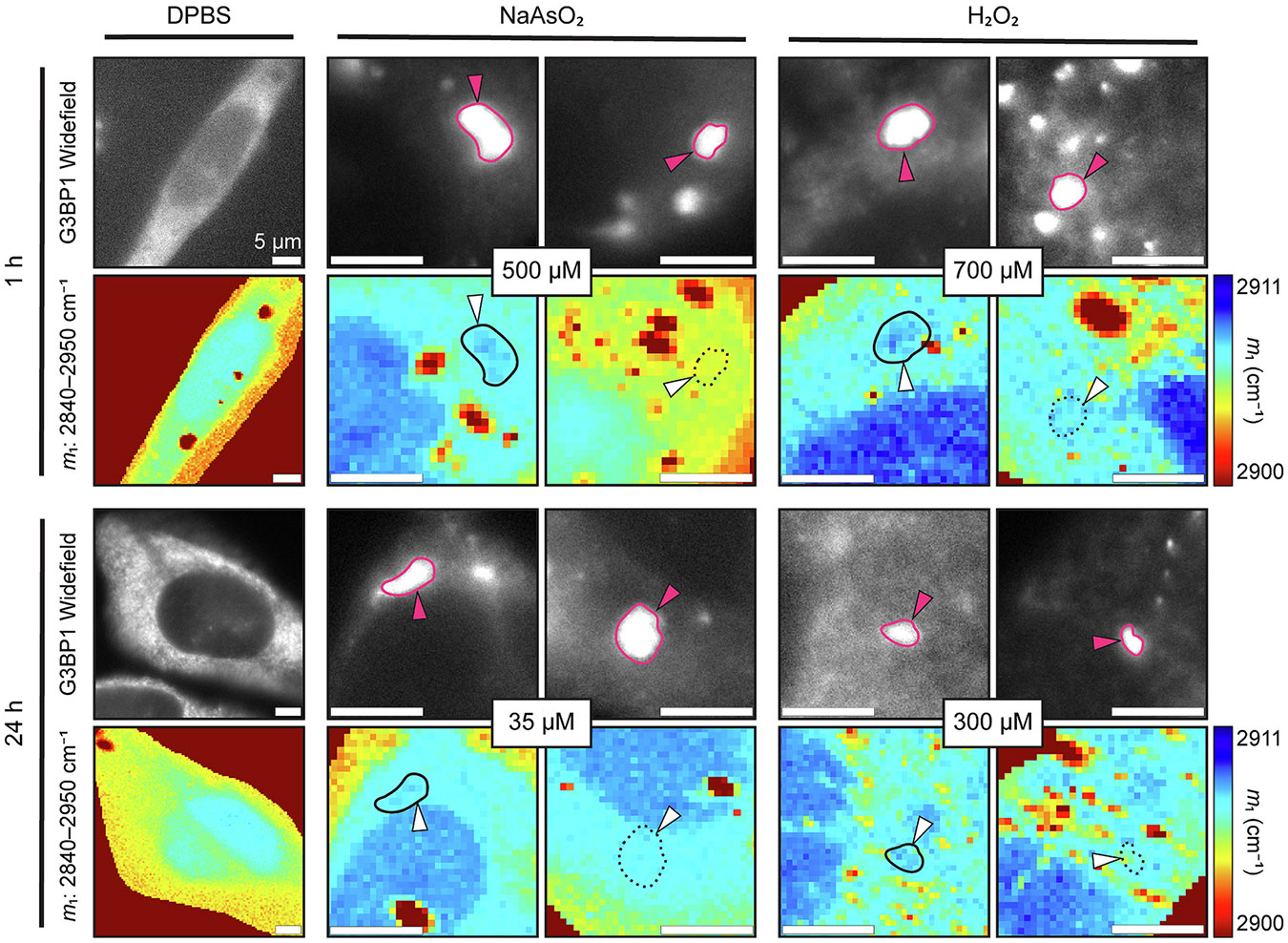
CRIFI reveals stress granules with different protein abundance between cells. U-2 OS cells treated with stressors at the indicated concentration and duration imaged by CRIFI. Widefield fluorescence images of anti-G3BP1 (WH0010146M1) antibody detected using Alexa405-labeled goat anti-mouse antibody and the correlated Raman maps generated by first moment $\left(m_{1}\right)$ analysis of the selected C─H stretch region (2840–2950 cm^−1^) are shown. Raman maps were collected using a 1.5 NA 60× oil immersion objective with 300-ms accumulation time per pixel with a 300-nm step-size per pixel using 50-mW laser power. For each stress condition, two types of G3BP1 fluorescent puncta are shown, ones that do (left, solid outline) and ones that do not (right, dashed outline) correspond to a protein-rich region in the Raman map. The *z*-plane for RSI in each image set was selected by performing a *z*-scan (an example is shown in Fig. S4) of the indicated punctum and picking the plane with maximum protein signal intensity as determined by the CH_3_ stretch band (2910–2950 cm^− 1^). Scale bars are 10 μm.

To improve the efficiency of CRIFI, we turned to performing widefield fluorescence imaging on the same microscope where Raman images are collected (Fig. 4). A fluorescence image is taken to provide a broad view of all the G3BP1-positive puncta within the cell. While there are many visible puncta by widefield fluorescence, they may not reside on the same focal plane. Thus, a single punctum of interest is selected (indicated by magenta outlines and arrowheads), and a *z*-scan is performed through the center of this punctum to determine the optimum *z*-plane for Raman mapping that corresponds to the plane with the highest protein density based on the integrated area of the methyl spectral region (2910–2950 cm^−1^). An example *z*-scan is shown in Fig. S4.

**Fig. 5.**
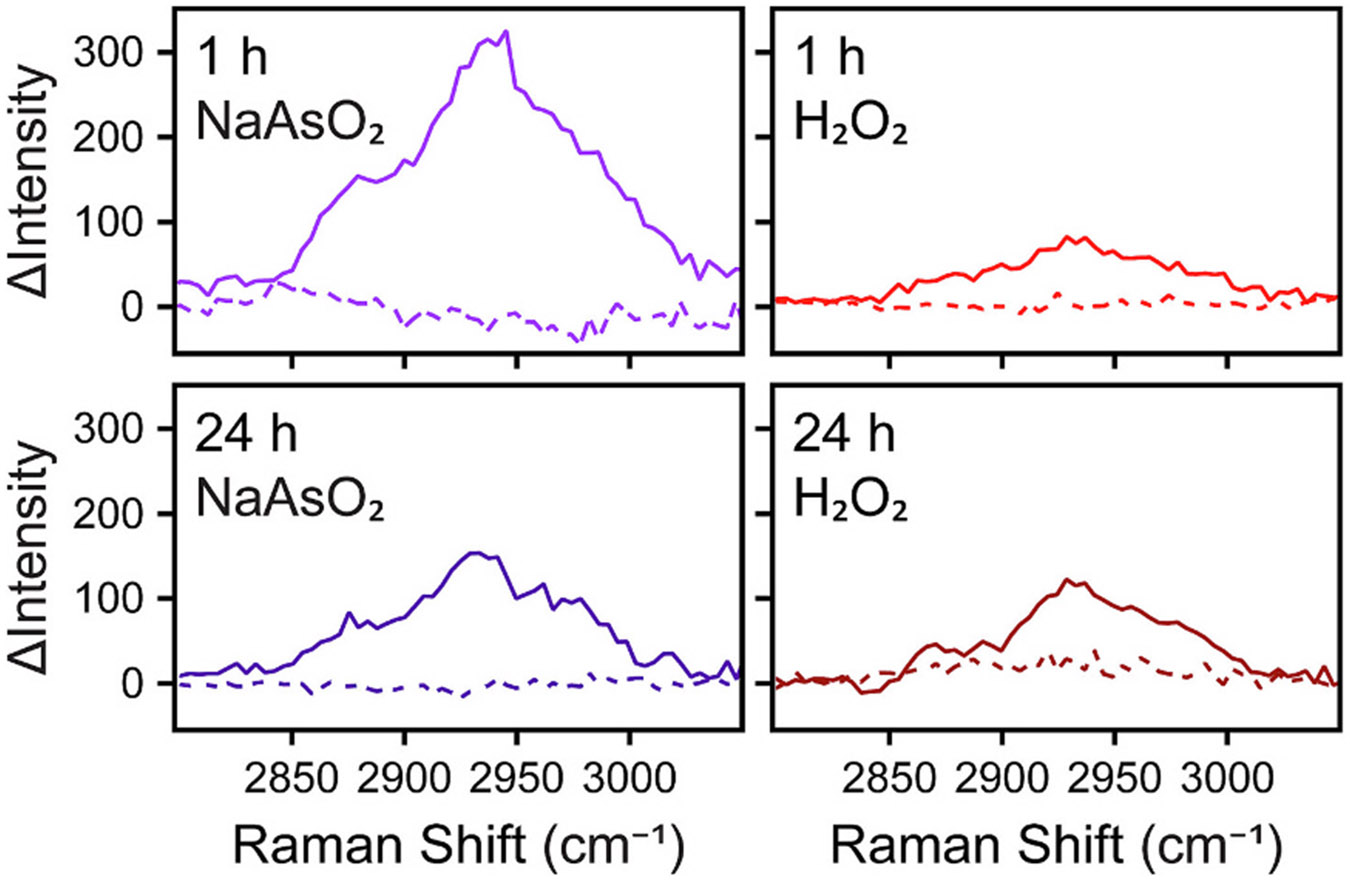
Difference Raman spectra corroborate increased protein content in a subset of SGs. Difference Raman spectra of ROIs in cells shown in Fig. 4. In each indicated condition, solid and dashed lines correspond to the puncta with solid and dashed outlines. Difference spectrum is calculated as the average spectrum of the punctum ROI minus the average spectrum of an equally-sized cytosolic ROI adjacent to the punctum. Protein content corresponds to the increase in CH_3_ stretching band (2910–2950 cm^−1^) within the C─H stretching region (2800–3050 cm^−1^).

**Fig. 6.**
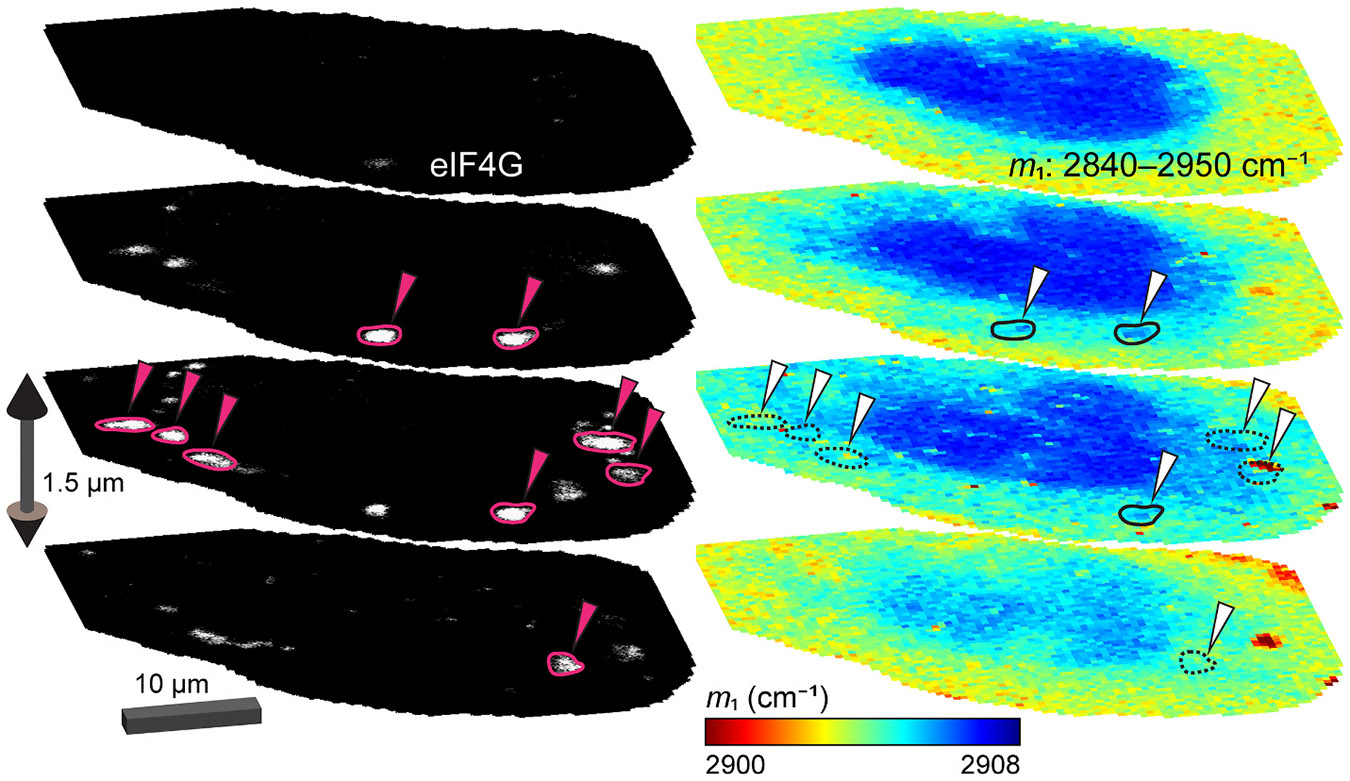
CRIFI reveals stress granules with different protein abundance within a single cell. 3-Dimensional CRIFI of a U-2 OS cell treated with 500 μM sodium arsenite for 30 min. Immunofluorescence (left) of anti-eIF4G (PA5-83016) antibody detected by Alexa405-labeled goat anti-rabbit secondary antibody. Correlated Raman maps (right) generated by first moment $\left(m_{1}\right)$ analysis of the selected C─H stretch region (2840–2950 cm^−1^) were collected using a 1.42 NA 60× oil immersion objective with 100-ms accumulation time per pixel with 400-nm step per pixel using 100-mW laser power. Solid outlines on the Raman maps denote puncta that are positive for both eIF4G and increased protein density. Dashed outlines denote puncta that are positive for eIF4G labeling but do not have a distinct protein accumulation on the Raman map. *Z*-slices are taken every 1.5 μm. Scale bar as indicated. Additional examples are shown in Fig. S5-S7.

Interestingly, in all four treatments, puncta were observed in which protein either was (solid outline) or was not (dashed outline) accumulated relative to the cytosol surrounding the punctum of interest as determined by $m_{1}$ analysis (Fig. 4). Accumulation or lack thereof is further confirmed by comparing intensity of the CH_3_ stretch (2910–2950 cm^−1^) for spectra in the outlined ROIs to the cytosol of each cell imaged (Fig. 5). Since the *z*-plane for each cell was specifically selected to align with the maximum protein density of this punctum, it may seem perplexing to find a lack of enriched protein concentration. However, if the puncta do not have an increase in protein accumulation that surpasses our detection limit, then the *z*-scan taken beforehand would not have been able to identify the true position of the punctum. It is therefore possible in these instances that the *z*-plane mapped by RSI is not necessarily aligned with the punctum but rather reflects the maximum cytosolic protein density. To account for this possibility, we performed CRIFI with an Evident FV4000 confocal laser scanning microscope before transferring to the Raman microscope and visualized multiple *z*-slices by each imaging modality (Figs. 6, and S5-7). For the cell shown, two distinct protein-rich puncta can be observed in the cytosol by RSI (solid outlines). These puncta colocalize with the SG-associated protein eIF4G, but there are several other fluorescent puncta throughout the cell for which there is no corresponding punctum on the Raman map (dashed outlines). With this complete view of the puncta across a single cell, we interpret discrepancies between the significant number of puncta visible by IF and the lack thereof with protein accumulation in the Raman maps to reflect a real difference between SGs. This implementation of CRIFI is the most robust for characterizing puncta with either phenotype, but clearly more work is needed in the future to determine how prevalent this observation is overall.

## 3. Discussion

SG formation in mammalian cells is generally accepted as a protective cellular response in the face of chemical assaults that generate oxidative stress. Additionally, these phase-separated condensates are believed to be a highly concentrated, dense phase composed of proteins and other biomacromolecules. However, the extent to which the composition of SGs is modulated by the specific stressor and how it may change with the duration of stress is not currently well known. Herein, we developed CRIFI to probe the distribution of biomacromolecules within and surrounding SGs formed under several oxidative stress conditions. Interestingly, Raman maps correlated with IF of G3BP1, a SG-protein, revealed that, in both acute and chronic treatments of NaAsO_2_ and H_2_O_2_, only a subpopulation of SGs exhibit protein accumulation, an unexpected and new result.

Differences between phenotypes of G3BP1-puncta following the applied stress treatments were, however, observed by biochemical assays. Notably, compared to the standard, acute NaAsO_2_ treatment, acute treatment with H_2_O_2_ and chronic treatment with either NaAsO_2_ or H_2_O_2_ produced far fewer cells that had puncta positivity (Fig. 2D). Furthermore, both durations of H_2_O_2_ treatments produced cells with more puncta than either NaAsO_2_ treatment (Fig. 2E), suggesting a variability in how the cell responds to oxidative stress from different sources. Still, the reduction in SGs in both chronic stress treatments is particularly confounding given that these conditions experienced both greater ROS accumulation (Fig. 1A) and a greater activation of antioxidant pathways (Fig. 1B). This and the low amount of ROS accumulation and antioxidant activity in the acute arsenite treatment, which is highly successful at inducing SGs, suggest that SG-induction is not directly associated with the intensity of the assault under oxidative stress conditions.

The discrepancy between the amount of oxidative stress and the incidence of SG-induction is likely due to multiple antioxidative responses being mounted simultaneously in cells, such that, even if SG nucleation occurs, their functionality and prolonged existence are not strictly necessary to avoid cell death. This has been shown to be possible as cells are capable of avoiding apoptosis even when key SG proteins are damaged by ROS [58]. In instances such as this, SGs may either never surpass the size necessary to be detected or disassemble prior to imaging, resulting in the less-than-100 % induction rate despite significant oxidative damage being incurred. The high degree of success for acute arsenite treatment in inducing puncta with SG-markers therefore indicates that the SG-induction pathway is rapidly and completely activated in this condition to a degree that renders additional responses to ROS less prevalent. On the other hand, SG-formation cannot be efficiently achieved by chronic exposure to a lower dosage of arsenite. Hydrogen peroxide treatments may be similarly ineffective at producing puncta with SG-markers due to favored activation of competing cellular antioxidants.

Our development of CRIFI exemplifies how the two techniques are particularly well suited for combination and how each benefits from being correlated with the other. In the cell imaged in Fig. 3, four distinct puncta are visible with increased protein concentration as determined by integration of the CH_3_ stretching band. Given the expected SG phenotype, one might assume from the Raman map alone that all of the puncta are, in fact, SGs. However, in the immunofluorescence image, we see that only three (white arrowheads) of these four are positively labeled for both G3BP1 and TDP-43. The fourth (magenta arrowhead) does not have detectable G3BP1 and is therefore more likely to represent a structure distinct from SGs, possibly reflecting a TDP-43-containing condensate or aggregate. For our purposes, CRIFI also significantly decreased the amount of time required to collect Raman maps because time was not spent mapping cells that did not have the desired phenotype.

Previously, SGs have been suggested to be low-density condensates [40] which may not even be dense in proteins [39]. This is surprising given that, as proteins phase separate, the structures that form should inherently have a greater protein concentration than their surroundings in order to achieve an isolated phase, yet, until now, this had not been observed. Using CRIFI to probe each of the stress conditions, we find the macromolecular contents of SGs are not so definitive. We do observe structures colocalizing with G3BP1 devoid of any apparent increase in protein abundance relative to the surrounding cytosol, corroborating previous studies [39,40], but it was surprising to also find G3BP1-positive puncta that *did* have increased protein levels (Figs. 4-6, and S5-7). This finding has multiple implications: firstly, it is possible that there is a discrepancy between what is commonly qualified as a SG by IF alone and what truly meets the expectations of a functional SG. Further investigation is needed to confirm this is indeed the case. Moreover, it would be pertinent to examine when or why this variance arises. Secondly, if SGs do serve as nucleation sites for cytotoxic protein aggregation, the underlying cause for some puncta to have protein accumulation while others do not needs to be elucidated to determine the association with disease progression. Regardless, the existence of a measurable difference between SGs at all, especially observed within a single cell, suggests that there are further mechanisms governing SG composition that are yet to be revealed and, importantly, could not be determined by IF imaging alone. As such, the correlative chemical information yielded by the addition of RSI to standard identification by IF proves invaluable for the study of SGs.

The apparent commonality of identifying puncta with and without protein accumulation following the four different treatments is in itself interesting. It suggests that this is an intrinsic trait of SGs generated under oxidative stress and is not the result of any one induction, despite each producing unique SG phenotypes as determined by IF. It therefore warrants additional characterizations of SGs to quantitatively determine if there are stressors that are more prevalent in producing puncta with protein accumulation. Further development of CRIFI to improve sensitivity will also prove useful in this endeavor, as the ability to detect the appearance of β-sheet in cells under select stressors could elucidate any potential role that these protein-rich puncta may have in amyloid formation.

In summary, this study introduces CRIFI, a new methodology for interrogating macromolecular distributions of endogenous complexes associated with target proteins, and this technique revealed novel features of SGs induced by oxidative stress. Further investigation into the mechanistic underpinnings of these characteristics may be pertinent to therapeutic interventions in neurodegenerative disorders and other diseases related to oxidative stress. CRIFI has a wide range of applications given the generality of secondary antibodies for detection of targets selected for labeling. To further expand the efficacy and capability of this technique, we envision the development of a dual fluorescent (*e.g.* FP fusion or dye-labeling) and Raman-active nanobody that has biorthogonal vibrations (carbon─deuterium (C─D) stretches [59] and carbon─carbon triple bonds (C≡C) [60,61]) in the cellular quiet region of the Raman spectrum. With generalizability and advantages as demonstrated here, we anticipate that there are many other systems that would benefit from this correlative imaging.

## 4. Materials and methods

### 4.1. Cell culture and stress induction

U-2 OS cells (ATCC cat. no. HTB-96) were maintained at 37 °C under a 5 % CO_2_ atmosphere in vented flasks containing McCoy’s 5 A medium (ATCC cat. no. 30–2007) supplemented with 10 % fetal bovine serum (ATCC cat. no. 30–2020) and 1 % penicillin/streptomycin (Gibco cat. no. 15140122). Upon reaching 80 % confluency, cells were incubated with 0.25 % trypsin-EDTA (ATCC cat. no. 30–2101) for 8 min at 37 °C and collected by centrifugation (500 rcf for 5 min). Pellets were then resuspended in fresh McCoy’s 5A media. For imaging experiments, cells were seeded at a density of 12,500 cells/cm^2^ into each compartment of a 4-chamber, 35 mm, #1.5 glass bottom dish with 20 mm well (Cellvis cat. no. D35C4–20-1.5-N) precoated with poly-d-lysine (Gibco cat. no. A3890401). For ROS detection and western blot analysis, cells were seeded at a density of 12,500 or 25,000 cells/cm^2^, respectively, in 6-well plates (Corning Falcon cat. No 353046) precoated with poly-d-lysine. Cells were allowed to adhere overnight (~19 h) at 37 °C in a 5 % CO_2_ atmosphere incubator before the addition of stress inducers. NaAsO_2_ solution (Sigma cat. no. 1.06277), freshly diluted H_2_O_2_ solution (Sigma cat. no. H1009), or sterile-filtered Dulbecco’s Phosphate Buffered Saline (DPBS, ATCC cat. no. 30–2200) treatments were added to sample media the following morning, and cells were incubated at 37 °C for the duration of the treatment as specified.

### 4.2. Reactive oxygen species quantification

The total amount of oxidative species generated was quantified by the conversion of 2′,7′-dichlorodihydrofluorescein diacetate (DCFH-DA, Sigma cat. no. D6883) into fluorescent 2′,7′-dichlorofluorescein (DCF) using an adapted protocol from Kim and Xue [62]. Following chemical treatment, media was removed, and cells were washed once with fresh, prewarmed media. DCFH-DA was diluted 1:1000 from a freshly made 10 mM stock down to 10 μM into prewarmed media, and the cells were incubated in this solution for 30 min at 37 °C. DCFH-DA-containing media was then removed, and cells were washed once with fresh media and twice with DPBS. Cells were lysed with prechilled RIPA buffer [25 mM Tris (Sigma cat. no. T5941), 150 mM NaCl (Sigma cat. no. S7653), 1 % Triton X-100 (Sigma cat. No 93426), 0.25 % SDS (Sigma cat. no. L3771), pH 8.0] on ice for 5 min. Adhered cell fragments were collected by scraping, and whole lysate was collected. Lysate was centrifuged at 16,100 rcf for 10 min at 4 °C, and the supernatant was collected and transferred to a 96-well plate (VWR cat. no. 10062–900) for fluorescence detection. Fluorescence was quantified on a Tecan Spark Multimode Microplate Reader using an excitation wavelength of 485 nm and an emission wavelength of 530 nm. Each treatment condition was plated in triplicate, and a total of five biological replicates were measured. A Bradford assay (Thermo Scientific cat. no. 23246) was performed from the remaining supernatant to determine total protein concentration, which was used to normalize fluorescence intensities.

### 4.3. Western blot

Cells were washed and lysed as described above. A Bradford assay was used to determine protein concentration in the samples, and 15 μg of each was resolved by SDS-PAGE (NuPAGE Bis-Tris 4–12 % gel, Invitrogen cat. no. NP0321BOX). Protein was transferred to a 0.45 μm PVDF membrane (Invitrogen cat. no. LC2005) using an Invitrogen XCell II Blot Module following the manufacturer protocol. Membranes were fixed with 4 % paraformaldehyde (PFA, Electron Microscopy Sciences cat. No 15710) for 30 min at RT, washed once with water, stained with Ponceau-S (MP Biomedical cat. no. 190644) for 5 min at RT, and again washed with water. Images of stained membranes were acquired in a lightbox. Membranes were incubated with blocking buffer (5 % blotting-grade blocker (Bio-Rad cat. no. 1706404) in TBS-T) for 1 h at RT. Primary antibody binding was performed in fresh blocking buffer containing 1:1000 rabbit anti-Nrf2 antibody (Abcam cat. no. ab137550) overnight at 4 °C. Membranes were washed four times with TBS-T for 5 min each before incubation with 1:10,000 goat anti-rabbit secondary antibody (Abcam cat. no. ab97080) in blocking buffer for 1 h at RT. Membranes were washed four times with TBS-T and once with water. Membranes were placed in an Amersham Hypercassette autoradiography cassette and coated with enhanced chemiluminescence (ECL) substrate (Thermo Scientific cat. no. 32134) for 5 min. Excess substrate was wicked off using filter paper, and luminescence was captured on CL-XPosure films (Thermo Scientific cat. no. 34090) in the Hypercassette for 90 s before being developed. Films were scanned and imported into ImageJ to enhance contrast.

### 4.4. Immunofluorescence

Cells were washed three times with DPBS prewarmed to 37 °C, then fixed with prewarmed 4 % PFA in DPBS at 37 °C for 15 min, and washed three times with DPBS at RT. Fixed cells were permeabilized with 0.5 % Triton X-100 in blocking buffer (3 % bovine serum albumin (Pentex cat. no. 81-003-2) in DPBS) for 7 min at RT, followed by three DPBS washes, and incubated for 1 h in blocking buffer at RT. After blocking, cells were washed once with DPBS and incubated with primary antibodies [mouse monoclonal anti-G3BP1 (Sigma cat. no. WH0010146M1), rabbit monoclonal anti-TDP-43 (4R5L7, Invitrogen cat. no. MA5-35273), rabbit monoclonal anti-TDP-43 (EPR5811, Abcam cat. no. ab133547), and rabbit polyclonal anti-eIF4G (Invitrogen cat. no. PA5-83016)] in blocking buffer for 1 h at RT. For 3-color imaging, rabbit anti-TDP-43 and rabbit anti-eIF4G antibodies were conjugated following the manufacturer protocol with CF647 (Sigma cat. no. MX647S100) and CF488 (Sigma cat. no. MX488AS50) dyes, respectively. Samples were washed three times with DPBS and incubated with secondary antibodies [goat anti-mouse A405 (Invitrogen cat. no. A-31553) and goat anti-rabbit A647 (Invitrogen cat. no. A-21235)] in blocking buffer for 1 h at RT. Labeled cells were washed three times with DPBS and were stored at 4 °C in the dark until imaging. Fluorescent images of two-color samples for SG counting and correlative imaging were acquired on an Evident FLUOVIEW FV4000 confocal laser scanning microscope using a 1.42 NA 60× oil objective (UPLXAPO60XO, Olympus). Fluorophores were excited with Coherent OBIS 405 and 561 nm lasers, and emission was detected at 400–550 and 620–750 nm, respectively. Three-color images were acquired with a 1.4 NA 63× oil objective on a Zeiss LSM 980 scanning confocal microscope (NHLBI Light Microscopy Core) using 405, 488, and 639 nm laser excitation, and emission was detected using optics as described in Table S1. Super-resolution images were acquired using the Airyscan 2 functionality on this instrument and were resolved automatically using the Airyscan Processing feature in the Zeiss Zen 3.8 software. Fluorescence images were imported into ImageJ for processing. For super-resolution images, masks were generated for each channel by calculating a threshold using Otsu’s method on Gaussian-blurred images after background subtraction.

### 4.5. Fluorescence spectroscopy

Fluorescence excitation and emission spectra were acquired on a Horiba Jobin Yvon Fluorolog FL-3 fluorimeter using a 3 × 3 × 10 mm quartz cuvette. Excitation spectra were recorded from 300 to 500 and 500 to 700 nm (1 nm step, 0.5 mm slit width, 0.5 s integration time) at the emission maximum of 420 and 660 nm (0.5 mm slit width) for Alexa405 and Alexa647, respectively. Emission spectra were recorded from 365 to 600 and 615 to 800 nm (1 nm step, 0.5 mm slit width, 0.5 s integration time) using excitation wavelengths of 350 and 600 nm (0.5 mm slit width) for Alexa405 and Alexa647, respectively. Emission intensity was corrected for lamp power and detector sensitivity.

### 4.6. Correlative Raman and immunofluorescence imaging

Immunofluorescence imaging was performed as described directly after immunolabeling of fixed cells. RSI was then performed on a home-built Raman microscope [63] using 488 nm laser excitation (Coherent Sapphire SF NX 488–100). The beam was collimated through a zoom beam expander (ZBE1A, ThorLabs), adjusted to match the back aperture of a high NA 60× oil objective (UPLAPO60XOHR or UPLXAPO60XO, Olympus), and directed into an Olympus IX-71 inverted microscope. The filter cube contained a 488 nm cleanup excitation filter (LL01–488-25, Semrock), a 488 nm dichroic (LPD02–488RU-25 × 36 × 1.1, Semrock), and a 488 nm ultrasteep long-pass edge emission filter (LP02-488RU-25, Semrock). Backscattered light is focused through a tube lens (M220, Horiba) into an iHR320 spectrometer (Horiba) with the entrance slit set to 50 μm. Light was dispersed using a 600 mm^−1^ diffraction grating onto a Horiba Symphony II BIDD CCD detector (1 MHz, best dynamic range, and binned to pixels 124–132 in the *y*-dimension for confocal resolution). The sample was scanned using a software-controlled stage (SCAN IM 120 × 80, Märzhauser-Wetzlar) and focus drive (MA42, Märzhauser-Wetzlar). Map step sizes (250–400 nm) and dwell time (100–300 ms) are as specified. The vertical step size for *z*-stacks was 1.5 μm. Camera, spectrometer, CCD detector, stage, and focus drive were controlled through Labspec 6.7.1 software (Horiba). Daily spectral calibration was performed using a linear correction of cyclohexane (10 × 0.5-s accumulations) to the ASTM E1840-96R22 standard values [64]. To obtain widefield images on the same microscope prior to map acquisition, a 405 nm laser (Coherent OBIS, 5 mW) is directed through a zoom beam expander (ZBE4A, ThorLabs) and focused into the back aperture of the objective. A 405 nm long-pass dichroic (Di02-R405–25 × 36, Semrock) was used to direct the excitation beam to the sample, and widefield images were acquired on an INFINITY 3-6URM CCD (Teledyne Lumenera) after passing through a 420 nm long-pass filter (BA420, Nikon). The following number of cells were imaged for each condition: [DPBS: *n* = 4 (1 h), *n* = 3 (24 h); NaAsO_2_: *n* = 9 (1 h); *n* = 7 (24 h); H_2_O_2_: *n* = 7 (1 h), *n* = 6 (24 h)]. Raman data were processed using MATLAB 2024b (Mathworks). Raman spectra were processed by subtracting a constant offset, and maps were generated by integrating the area $\left(I_{a}\right)$ or calculating the first moment $\left(m_{1}\right)$, which is the weighted mean frequency, of the specified spectral region.

## Supplementary Material

1

## Data Availability

All data associated with this study have been deposited in Figshare at https://doi.org/10.25444/nhlbi.30032155.

## Appendix A. Supplementary data

Supplementary data to this article can be found online at https://doi.org/10.1016/j.jinorgbio.2025.113091.

## Abbreviations:

ROS: reactive oxygen species
SG: stress granule
RSI: Raman spectral imaging
IF: immunofluorescence
CRIFI: correlative Raman and immunofluorescence imaging
NaAsO_2_: sodium arsenite
H_2_O_2_: hydrogen peroxide
G3BP: Ras GTPase-activating protein-(SH3 domain)-binding protein
TDP-43: transactive response DNA binding protein of 43 kDa
hnRNP A1: heterogeneous nuclear ribonucleoprotein A1
FUS: fused-in sarcoma
eIF4G: eukaryotic initiation factor 4G
eIF2^α^: eukaryotic initiation factor 2^α^
TIA-1: T-cell intracellular antigen 1
PABP-1: poly(A)-binding protein 1
Keap1: Kelch-like ECH-associated protein 1
Nrf2: nuclear factor erythroid 2-related factor 2
FP: fluorescent protein
DPBS: Dulbecco’s phosphate buffered saline
PFA: paraformaldehyde
DCF: 2′,7′-dichlorofluorescein
DCFH-DA: 2′,7′-dihydrodichlorofluorescein diacetate
