# Supplementary material for: Correlative Raman and immunofluorescence imaging reveals different protein abundance between stress granules induced by oxidative damage
Source: J Inorg Biochem. Author manuscript; Available in PMC 2025 Dec 2. (PMC12666775; doi:10.1016/j.jinorgbio.2025.113091)
Supplement: 1 [file NIHMS2116443-supplement-1.pdf]

Supplementary Data for Correlative Raman and immunofluorescence imaging reveals different protein abundance between stress granules induced by oxidative damage by KL Gery, S Ramos & JC Lee

Table S1. Optical setup for detection of each channel in 3-color immunofluorescence imaging acquisition on the Zeiss 980 using Airyscan 2.

**Dye**	**Filter 1**	**Filter 2**	**Detection Window**
Alexa 405	SBS SP 505	Plate	200–505 nm
CF488	SBS SP 615	BP 495–555 + LP 660	495–555 nm
CF647	SBS LP 640	BP 420–550 + LP 605	640+ nm

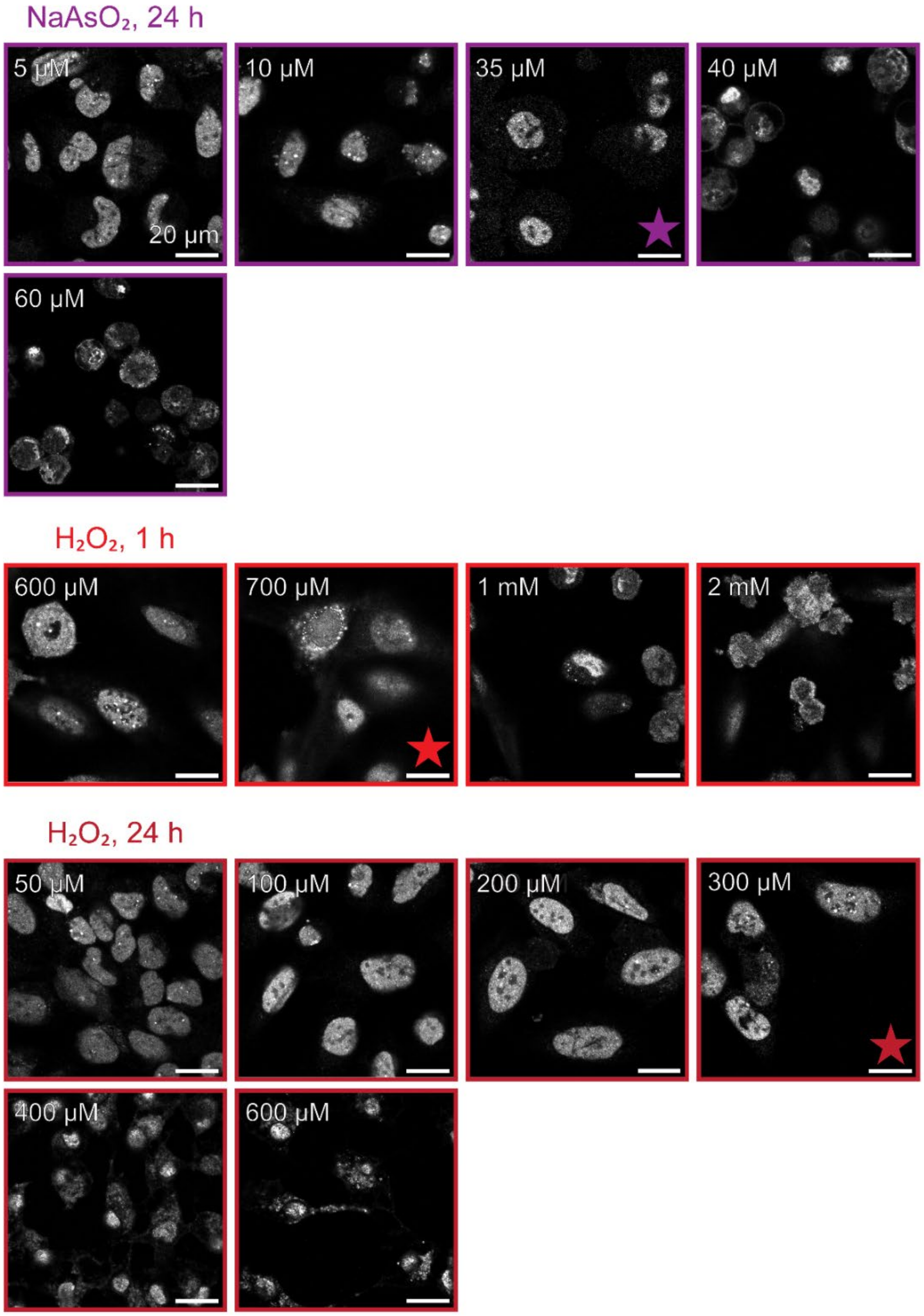

Fig. S1. Confocal fluorescence images of TDP-43 (anti-TDP-43, MA5-35273) in U-2 OS cells in the presence of increasing $NaAsO_2$ for 24 h or H_2O_2 for 1 and 24 h treatments. Stressor used, its concentration, and duration of treatment for each image are as indicated. Scale bars are 20 μm. Starred images correspond to selected concentrations used in this study.

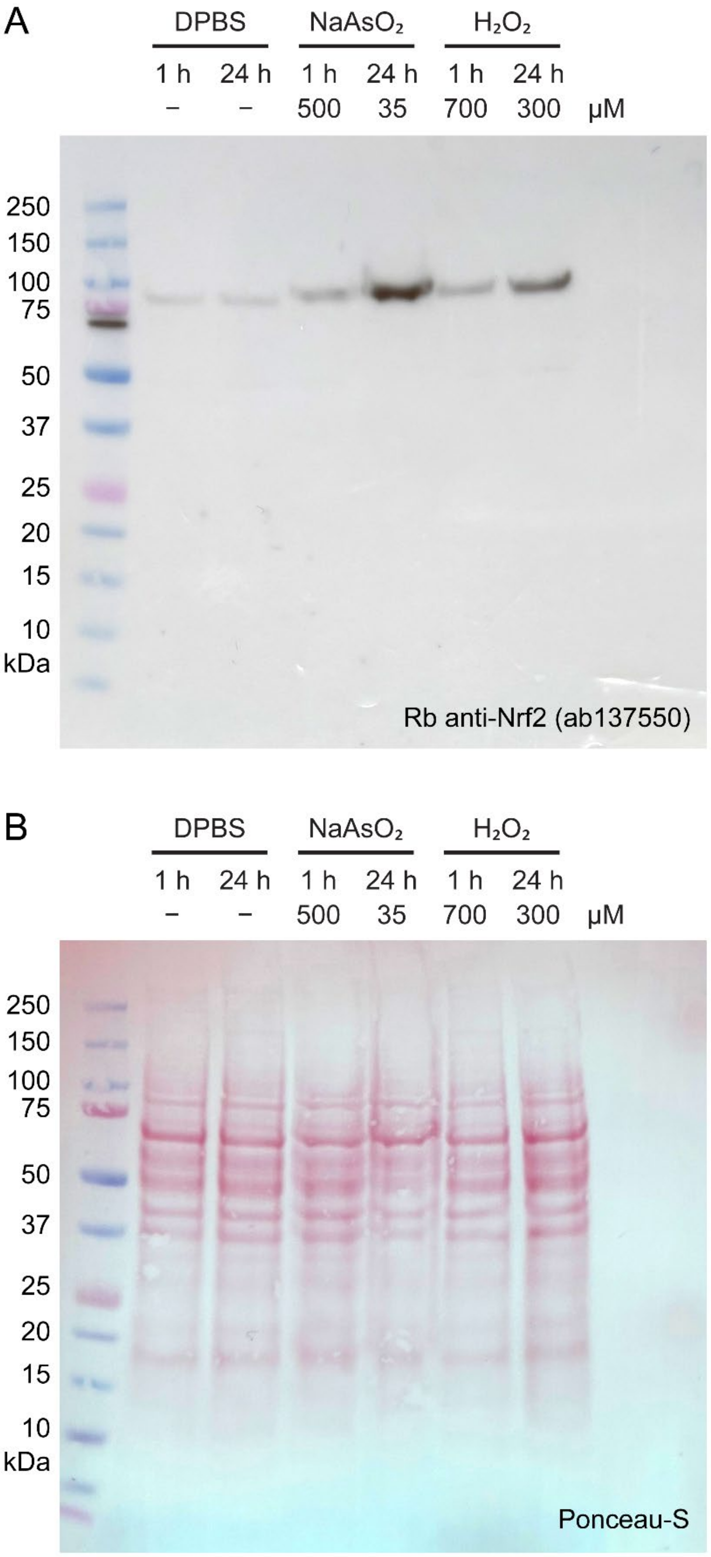

Fig. S2. Full views of the blots shown in **Fig. 1B**

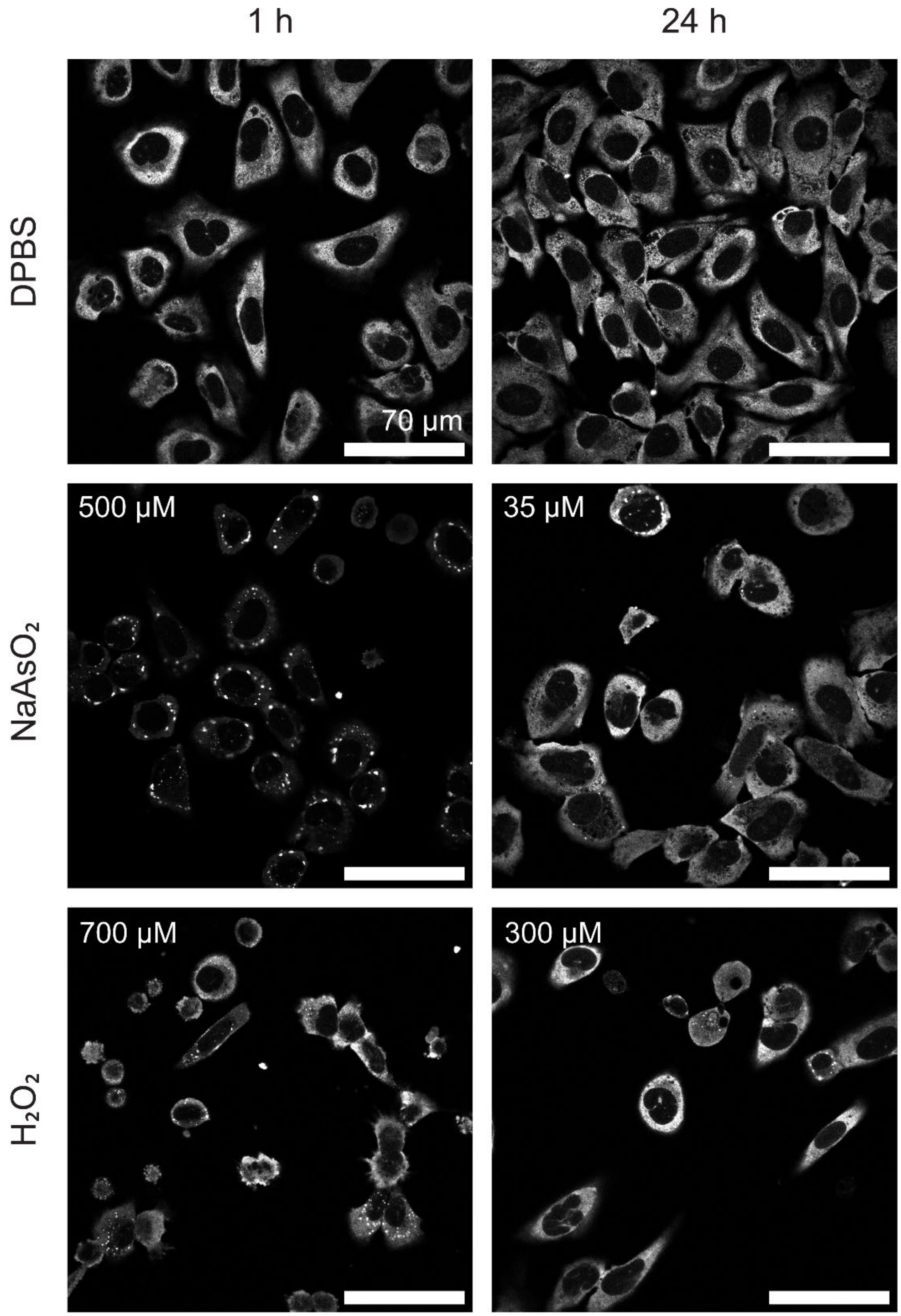

Fig. S3. Representative confocal fluorescence images of G3BP1 (anti-G3BP1, WH0010146M1) in U-2 OS cells used for SG-punctum counting. Stressor used, its concentration, and duration of treatment for each image are as indicated. Scale bars are 70 μm.

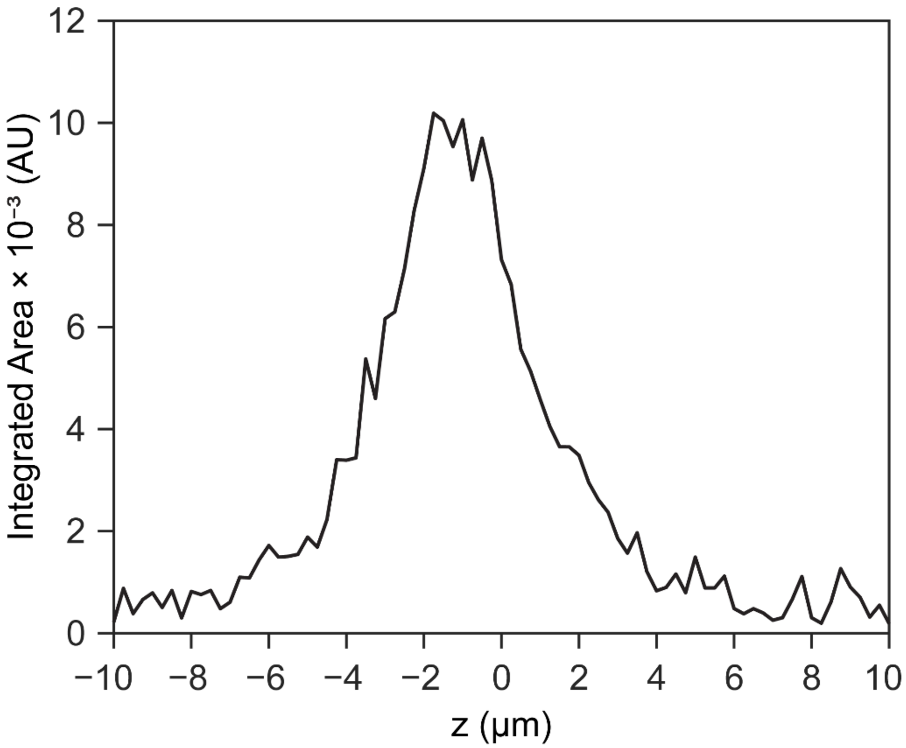

Fig. S4. Representative Raman *z*-scan through the center of a punctum that positively stained for anti-G3BP1 identified by widefield fluorescence. Protein content is evaluated by integrated area under the CH_3 stretch band (2910–2950 cm^{-1}) at each *z*-position (500-ms accumulation time for each step and 0.25-μm steps).

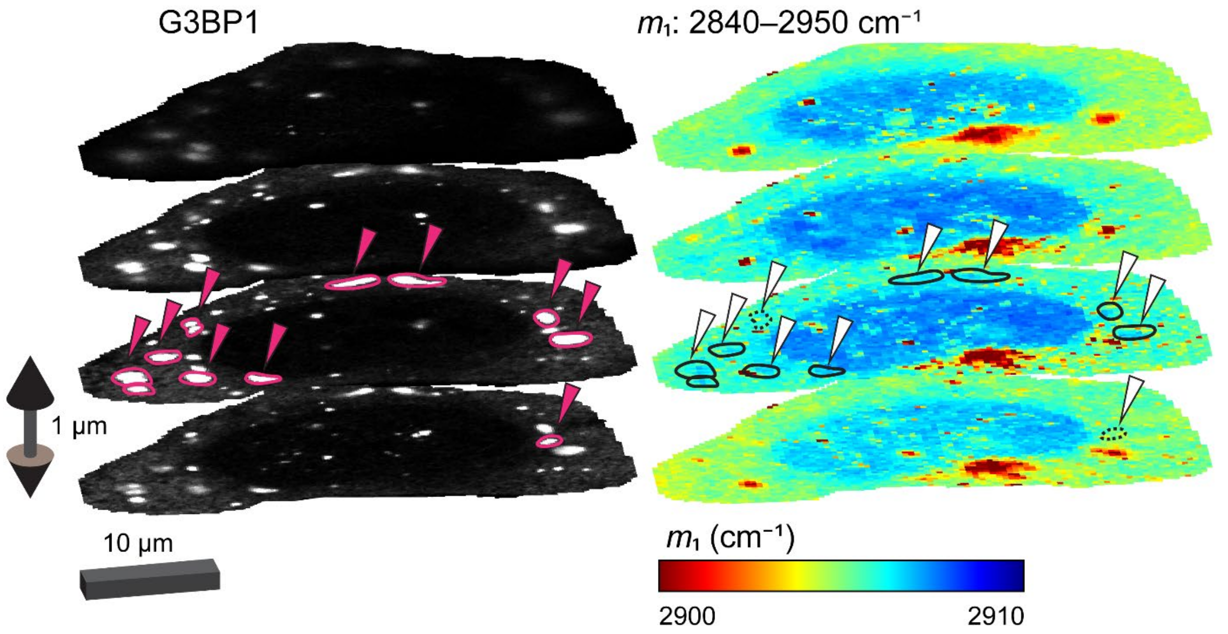

Fig. S5. Additional 3-dimensional CRIFI of a U-2 OS cell treated with 500 µM sodium arsenite for 30 minutes. Immunofluorescence (left) of anti-G3BP1 (WH0010146M1) antibody detected by Alexa405-labeled goat anti-rabbit secondary antibody. Correlated Raman maps (right) generated by first moment (m_1) analysis of the selected C–H stretch region (2840–2950 cm^{-1}) were collected using a 1.5 NA 60× oil immersion objective with 300-ms accumulation time per pixel with 500-nm step per pixel using 50-mW laser power. Solid outlines on the Raman maps denote puncta that are positive for both G3BP1 and increased protein abundance. Dashed outlines denote puncta that are positive for G3BP1 labeling but do not have a distinct protein accumulation on the Raman map. *Z*-slices are taken every 1 µm. Scale bar as indicated.

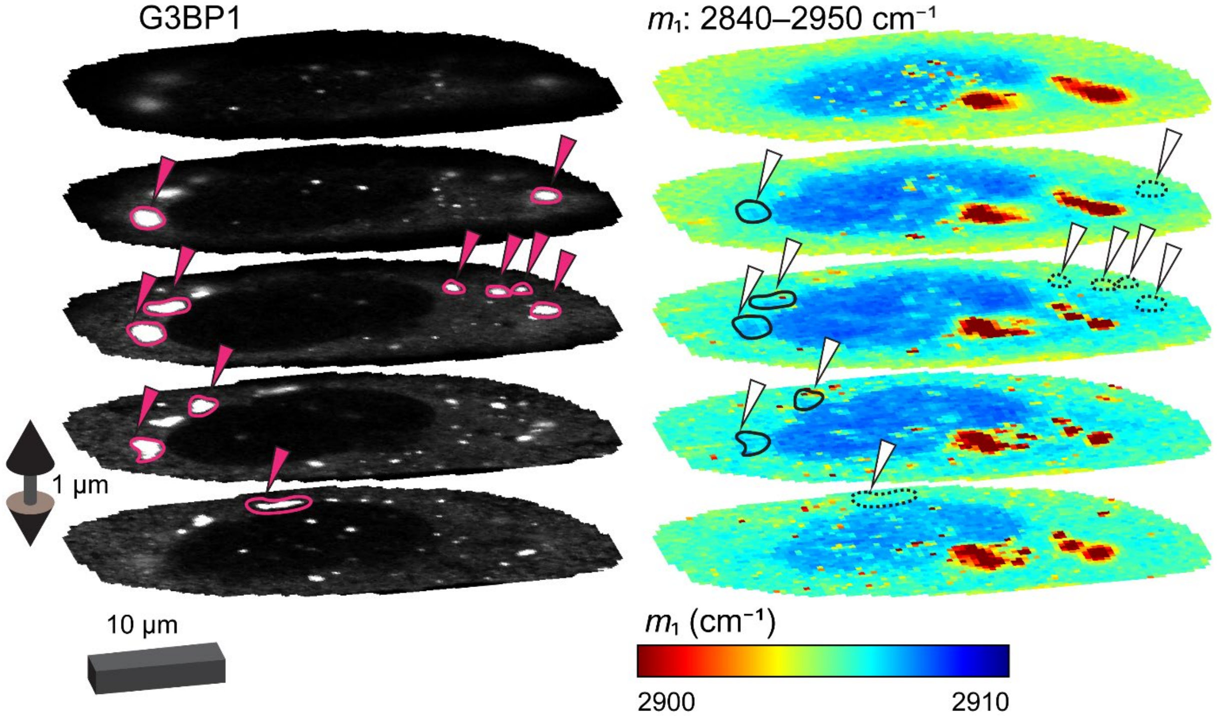

Fig. S6. Additional 3-dimensional CRIFI using the same conditions as in **Fig. S5**.

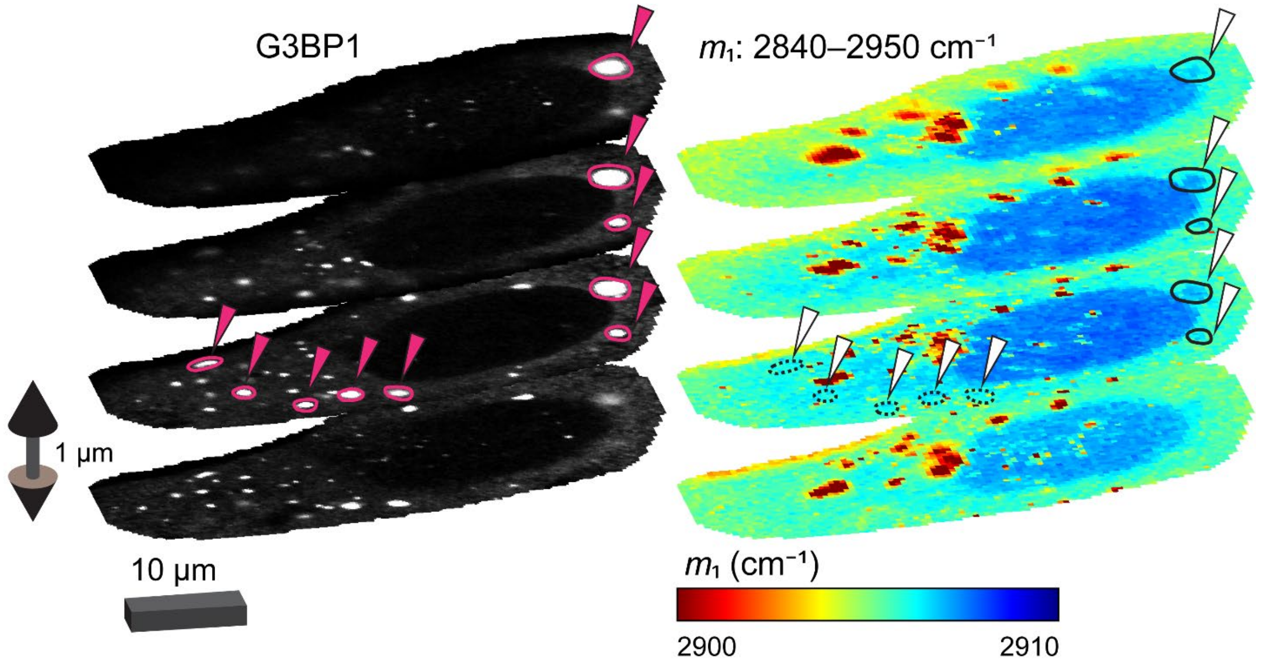

Fig. S7. Additional 3-dimensional CRIFI using the same conditions as in **Fig. S5**.
